# Thermal Inactivation Kinetics of Kudzu (*Pueraria lobata*) Polyphenol Oxidase and the Influence of Food Constituents

**DOI:** 10.3390/foods10061320

**Published:** 2021-06-08

**Authors:** Junping Liu, Jiayan Zhang, Tao Liao, Lei Zhou, Liqiang Zou, Yafei Liu, Li Zhang, Wei Liu

**Affiliations:** 1 State Key Laboratory of Food Science and Technology, Nanchang University, Nanchang 330047, China; ljpfood@163.com (J.L.); ncuzhangjiayan@163.com (J.Z.); 15180122056@163.com (T.L.); ncuskzhoulei@163.com (L.Z.); zouliqiang2010@163.com (L.Z.); ncuspyliuyafei@163.com (Y.L.); 2 Key Laboratory of Tropical Crop Products Processing of Ministry of Agriculture and Rural Affairs, Agricultural Products Processing Research Institute of Chinese Academy of Tropical Agricultural Sciences, Guangdong 524001, China; zhang1993123321@163.com; 3 National R&D Center for Freshwater Fish Processing, Jiangxi Normal University, Nanchang 330022, China

**Keywords:** polyphenol oxidase, kudzu, thermostability, thermal inactivation kinetics, food constituents, conformation

## Abstract

The thermal inactivation kinetics of kudzu (*Pueraria lobata*) polyphenol oxidase (PPO) were investigated in model and food systems. PPO in kudzu tissue (tPPO) showed a higher thermostability than that of PPO in crude extract (cPPO) and purification fractions (pPPO). The PPO inactivation rate constant (*k*) increased with an increase in temperature, and tPPO showed the lowest *k* value, followed by that of cPPO and pPPO at the same temperature, indicating that PPO in the food system was more resistant to thermal treatment. Food constituents (pectin, starch, sucrose, and bovine serum albumin) in the food system decreased the activity of PPO but increased the thermostability of PPO, among which pectin exhibited the strongest protective effect against thermal inactivation, and the influence of sucrose was much slighter than that of other macromolecules. Fluorescence emission spectra indicated that pPPO exhibited stronger interactions with pectin than sucrose, and pPPO with pectin showed a more stable conformation under thermal treatment.

## 1. Introduction

Kudzu (*Pueraria lobata*) is a herbaceous, perennial plant native to East Asia and belongs to the Leguminosae family. It is a rich source of starch, dietary fiber, minerals, and isoflavonoids and possesses many nutritional and pharmacological activities [1,2,3], such as antioxidant, anticancer, anti-inflammation, and neuroprotective properties. Additionally, kudzu is used as a vegetable and edible ingredient in the preparation of various foods [4]. For example, kudzu starch is used in the production of noodles, beverages, oral liquids, and various dishes, and it serves as a sauce thickener [5]. Food products containing components of kudzu have begun to attract interest from both consumers and researchers due to their health benefits. However, enzymatic browning of kudzu during storage, handling, and processing negatively affects its sensory and nutritional properties. Polyphenol oxidase (PPO) is the primary enzyme involved in enzymatic browning [6,7]. Therefore, the inactivation of PPO is important in improving the quality attributes of kudzu products.

Thermal treatment has been widely used as a non-chemical means for enzyme inactivation in food processing. The thermal inactivation of PPO from fruits and vegetables has been well evaluated in mushrooms [8,9], pear [10,11], apple [12,13], chestnut kernel [14], and pineapple [15]. The thermostability of PPO highly depended on the environmental conditions, and an obvious difference was observed in food and model systems. Purified apple PPO was almost completely inactivated after incubation at 65 °C for 20 min [13], while PPO in apple slices still retained about 40% relative activity in the same condition [12]. Compared to crude PPO extract from pear [11], PPO in pear puree [10] showed higher thermostability at a temperature range from 30 to 100 °C. The food system differs from the model system since the complex food constituents may affect the enzymatic properties during food processing [16]. The impact of pectin [17,18], protein [19,20], and sugar [21] on enzymatic properties have been investigated. However, there are few reports comparing the thermostability of PPO in food and model systems in the same study, and the effect of food constituents on the thermal inactivation kinetics remains unclear.

In this study, PPO from kudzu was purified and characterized. The thermal stability and inactivation kinetics of kudzu tissue PPO, crude PPO, and purified PPO were evaluated and compared with each other. To further illustrate the lower thermal inactivation effect in kudzu tissue, the content of food constituents, including pectin, protein, sucrose, and starch, in kudzu was analyzed, and the effect of these constituents on PPO activity, thermal inactivation kinetics, and conformation change was also evaluated.

## 2. Materials and Methods

### 2.1. Materials

Kudzu root (*Pueraria lobata*) was harvested from the plantation at Shangrao, Jiangxi province in eastern China, and stored at 4 °C until processing. Triton X-100, polyvinylpolypyrrolidone (PVPP), and *m*-hydroxydiphenyl were obtained from Aladdin Chemicals Co. (Shanghai, China). Catechol was obtained from Macklin Chemicals Co. (Shanghai, China). Pectin was purchased from Sigma-Aldrich Co. (Shanghai, China). Bovine serum albumin (BSA), starch, sucrose, D-Galacturonic acid, D-Glucose ammonium sulfate, tetramethylethylenediamine (TEMED), phenylmethylsulfonyl fluoride (PMSF), SDS-PAGE loading buffer, and standard protein mixture (Marker) were purchased from Solarbio Science and Technology Co. (Beijing, China). All the other chemicals were of analytical grade. Double-distilled water was used for preparing all the solutions.

### 2.2. PPO Extraction

The extraction and purification of PPO from kudzu were carried out according to the method of Liu et al. [22] with modifications. After washing and peeling, kudzu was sliced and mashed using a blender (HX-PB1053, AUX, Zhejiang, China) with 0.1 M phosphate buffer (pH 6.5) containing 2% (*w*/*v*) polyvinylpolypyrrolidone (PVPP), 0.5 M NaCl, and 1% (*v*/*v*) triton X-100 in a proportion of 1:1 (*v*/*v*) to yield puree. The mixture was stirred in an ice-water bath for 2 h and centrifuged (SORVALL Biofuge primo R, Thermo, Germany) for 20 min at 11,000× *g* at 4 °C. The supernatant was collected as the crude PPO (cPPO) and stored at 4 °C until use.

### 2.3. PPO Purification

To obtain purified PPO (pPPO), a two-step purification was conducted. Firstly, ammonium sulfate was added to cPPO, and the precipitate obtained between 20% and 30% saturation was collected by centrifugation at 11,000× *g* for 25 min at 4 °C. The precipitate was dissolved in phosphate buffer (pH 6.5) and dialyzed against the same buffer at 4 °C for 24 h. Then, the dialyzed fraction was loaded onto a DEAE Sepharose fast flow column (8 cm × 1 cm, GE, Healthcare Bio-Sciences AB, Sweden), which was pre-equilibrated with 50 mM phosphate buffer (pH 6.5). The column was eluted with a linear gradient of sodium chloride (0–0.5 M) in 50 mM phosphate buffer (pH 6.5) at a flow rate of 0.2 mL/min. SDS-PAGE electrophoresis was performed to determine purity [23].

### 2.4. Constituent Determination

Four main constituents (pectin, protein, starch, and sucrose) in kudzu tissue, the cPPO solution, and the pPPO solution were analyzed. The content of pectin was determined by *m*-hydroxybiphenyl and expressed as galacturonic acid equivalents [22]. Protein concentration was determined by the Bradford method [24], using bovine serum albumin as standard. Starch and sucrose contents were determined by acid hydrolysis and expressed as reducing sugar equivalents.

### 2.5. PPO Activity Assay

PPO activity was determined based on the method of Terefe et al. [25] with slight modifications. The reaction mixture consisted of 2.7 mL of McIlvaine buffer (pH 5.0), 0.2 mL of 0.4 M catechol solution, and 0.1 mL of PPO sample. The absorbance of the assay mixture was measured at 25 °C in the kinetic mode at 420 nm for 1 min using a UV-visible spectrophotometer (UV-1600PC, Mapada, Shanghai, China). The relative activities of PPO were calculated in accordance with Equation (1):

(1) $$\mathrm{Relative}\;\mathrm{activity}\left(\%\right)=\frac{\mathrm{Activity}\;\mathrm{of}\;\mathrm{treated}\;\mathrm{enzyme}}{\mathrm{Activity}\;\mathrm{of}\;\mathrm{untreated}\;\mathrm{enzyme}}\times 100\%$$

### 2.6. Sample Preparation

Before thermal treatment, kudzu slices (20 g) were vacuum packed in polyethylene bags (5 cm × 5 cm), while the cPPO and pPPO (0.2 mL) samples with 2.7 mL of McIlvaine buffer (pH 5.0) were filled in capillary tubes (5 mL). Samples were kept at 4 °C for 2 h to minimize heating lags.

The effects of food constituents on the activity of pPPO were examined, with pectin, BSA, starch, and sucrose in concentrations ranging from 0.5% to 3% (*w*/*v*), respectively. The resulting mixtures were labeled as “pectin-pPPO”, “BSA-pPPO”, “starch-pPPO”, and sucrose-pPPO”, respectively. They were stirred in an ice-water bath for 1 h and were immediately used in the experiment.

### 2.7. Thermal Processing

The thermal stability and thermal inactivation kinetics of PPO in kudzu tissue (tPPO), cPPO, pPPO, pectin-pPPO, BSA-pPPO, starch-pPPO, and sucrose-pPPO were tested according to the method of Tan et al. [26]. The thermal stability was measured by incubating samples at temperatures ranging from 45 to 90 °C at 10 min. Thermal inactivation kinetics was carried out at temperatures ranging from 50 to 80 °C for 0–60 min. After thermal treatment, all samples were rapidly cooled in ice-water, then the activity of the sample was determined.

The thermal inactivation kinetics of samples was described by the first-order kinetic reaction (Equation (2)):

(2) $$\ln(A_{t}/A_{0})=-kt$$

where *A*_0_ is the initial activity of the samples, *A_t_* is the residual activity after treatment time *t* (min), and *k* is the inactivation rate constant (min^−1^).

The Arrhenius law was used to describe the temperature dependence of the *k*-values and to estimate the activation energy by linear regression analysis (Equation (3)):

(3) $$\ln\left(k\right)=\ln\left(k_{0}\right)-\frac{E_{a}}{R\cdot T}$$

where *k*_0_ is the Arrhenius constant (min^−1^), *T* is the absolute temperature (K), and *k* is the inactivation rate constants at *T* (min^−1^). *E*_*a*_ is the activation energy (kJ/mol), and *R* is the universal gas constant (8.314 J/(mol·K)).

The thermodynamic parameters of inactivation (change in enthalpy (Δ*H*), Gibbs free energy (Δ*G*), and entropy (Δ*S*)) were determined using the equations presented below (Equations (4)–(6)):

(4) $$\Delta G=-R\cdot T\cdot \ln\left(\frac{k\cdot h_{p}}{K_{B}\cdot T}\right)$$

(5) $$\Delta H=E_{a}-R\cdot T$$

(6) $$\Delta S=\left(\frac{\Delta H-\Delta G}{T}\right)$$

where *R* is the universal gas constant (8.314 J/(mol·K)), *k* is the inactivation rate constant at *T* (s^−1^), *h_p_* is the Planck’s constant (6.6262 × 10^−34^ J·s), and *K_B_* is the Boltzmann’s constant (1.3806 × 10^−23^ J/K).

The half-life (*t*_1/2_) value of inactivation is given by Equation (7):

(7) $$t_{1/2}=\frac{\ln(2)}{k}$$

The decimal reduction time (*D* value) is the time needed to reduce the initial activity by 90%, given by Equation (8):

(8) $$D=\frac{\ln(10)}{k}$$

The *Z* value is the temperature needed to reduce the *D* value by one log cycle (temperature sensitivity parameter), and it is obtained by plotting the log *D* values versus the corresponding temperatures.

### 2.8. Fluorescence Spectra Analysis

Fluorescence emission spectra of pPPO, pectin-pPPO, and sucrose-pPPO were measured using a F-4500 spectrophotometer (Hitachi, Tokyo, Japan). The details are as follows: 0.6 mg/mL pPPO was added into quartz cuvettes and treated with 0, 0.2%, 0.4%, 0.6%, 0.8%, and 1.0% (*w*/*v*) pectin and sucrose to investigate their impact on the tertiary structure of pPPO. In addition, pPPO, pectin (0.4%)-pPPO, pectin (1.0%)-pPPO, and sucrose (1.0%)-pPPO were subjected to thermal processing at 50–80 °C to further explore the effect of pectin and sucrose on the conformation change against heat. As described by Zhou et al. [27], all the samples were excited at 280 nm and scanned from 420 to 300 nm at room temperature (25 ± 1 °C), with the excitation and emission slit bandwidths of 5 nm.

### 2.9. Statistical Analysis

All the experiments were performed at least in triplicate. The values are presented as means ± standard deviation (SD). The significant differences were determined based on an analysis of variance (ANOVA) with significance at 95% confidence.

## 3. Results and Discussion

### 3.1. Purification and Characterization of PPO from Kudzu

PPO from kudzu was purified by ammonium sulfate fractionation together with DEAE Sepharose fast flow column. PPO was successfully purified 38.4-fold with 32.9% recovery following DEAE Sepharose fast flow column (Supplementary Material Table S1). After conducting the SDS-PAGE electrophoresis (Supplementary Material Figure S1), only a single prominent protein band of approximately 21 kDa was obtained, which confirmed the effectiveness of this purification procedure. The optimum pH of PPO from kudzu ranged from 4.5 to 5.0, and the Michaelis–Menten constant (*K*_m_) and maximum velocity (*V*_max_) were estimated to be 23.54 mM and 0.37 ΔOD/min (catechol as substrate), respectively (Supplementary Material Figure S2). Moreover, the general constituents (starch, protein, pectin, and sucrose) in kudzu were determined. Starch showed the highest content (21.42%) in kudzu tissue (Supplementary Material Table S2). After extraction, the cPPO solution still contained a certain amount of starch, pectin, sucrose, and protein, but starch, pectin, and sucrose were no longer detected in the pPPO solution and the protein concentration was significantly reduced.

### 3.2. Thermal Stability of PPO

**Figure 1:**
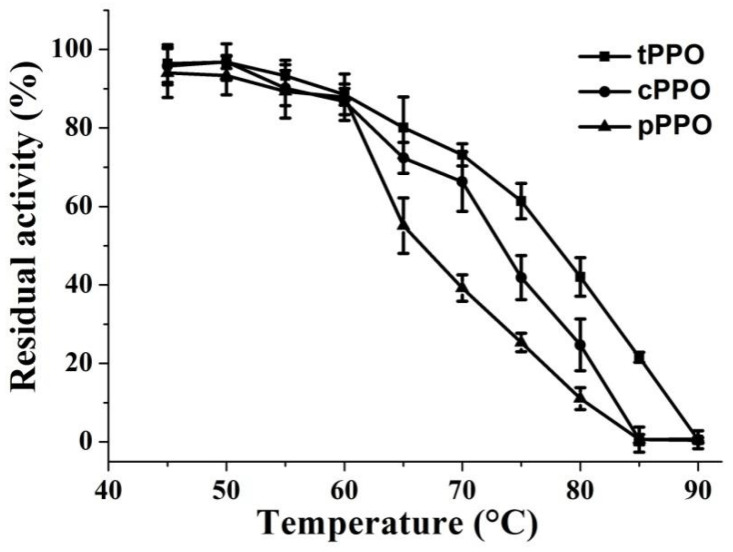
Thermal stability of PPO at temperatures ranging from 45 to 90 °C for 10 min. tPPO (■): PPO in kudzu tissue. cPPO (●): PPO from the crude kudzu extract. pPPO (▲): purified PPO from kudzu.

Thermal processing from 45 to 90 °C for 10 min resulted in different inactivation effects on kudzu tissue PPO (tPPO), crude PPO (cPPO), and purified PPO (pPPO) (Figure 1). The activity of tPPO, cPPO, and pPPO decreased gradually with increasing processing temperatures. After 10 min treatment at 45 to 60 °C, only slight inactivation was found, and the activity of tPPO, cPPO, and pPPO remained above 80% at 60 °C, which indicated that kudzu PPO exhibited high resistance to mild thermal processing between 45 and 60 °C. With the continuous increase in temperature, PPO activity declined sharply, and cPPO and pPPO were completely inactivated at 85 °C, while tPPO was completely inactivated at 90 °C. Between 60 and 90 °C, pPPO showed the fastest reduction rate in activity, followed by cPPO and tPPO. After 10 min treatment at 80 °C, the residual activity of pPPO was only 11.0%, whereas the values of cPPO and tPPO were 24.7% and 42.1%, respectively. These results illustrate that tPPO had the strongest thermostability while pPPO had the weakest. PPO in the food system displayed higher thermostability than that in the model system. This may be due to the effect of constituents in kudzu tissue.

In our previous studies, it was found that PPO in pear puree [10] showed higher residual activity than that of the partially purified PPO from pear [11] after thermal treatment in the temperature range of 30–100 °C. The results are consistent with the viewpoint in this study that PPO in the food system was more resistant to heat, and a reasonable explanation for this could be that food constituents such as proteins and carbohydrates can protect the zymoprotein from heat [28].

### 3.3. Thermal Inactivation Kinetics and Thermodynamics of PPO

**Figure 2:**
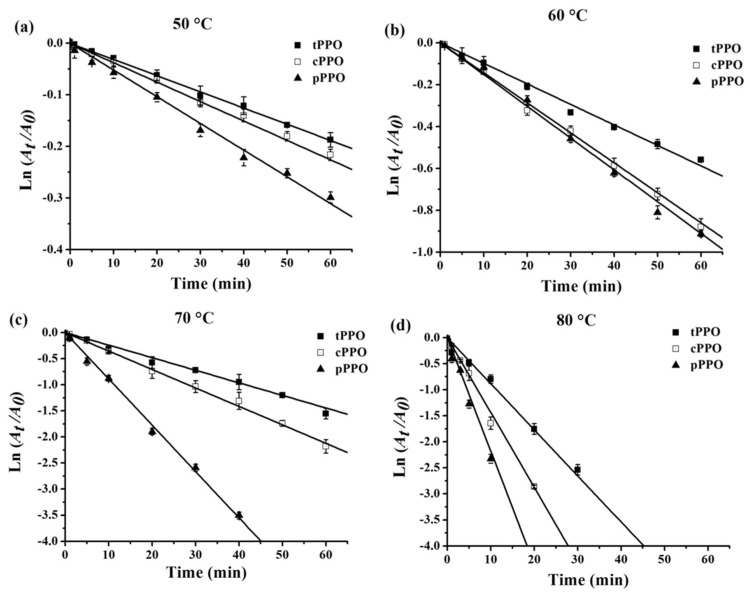
Thermal inactivation kinetics curves of PPO at 50 °C (**a**), 60 °C (**b**), 70 °C (**c**), and 80 °C (**d**) for 0–60 min. tPPO (■): PPO in kudzu tissue. cPPO (**□**): PPO from crude extract of kudzu. pPPO (▲): purified PPO from kudzu.

Based on the results of the thermal stability, the thermal inactivation of PPO was performed at temperatures that ranged from 50 to 80 °C and the incubation time ranged from 0 to 60 min (Figure 2). Lower activity of PPO was observed in all samples as a result of a higher temperature or longer incubation time. As shown in Figure 2a, when the temperature of thermal processing was 50 °C, the thermal inactivation of pPPO was the fastest, followed by that of cPPO, while that of tPPO was the slowest. The residual activities of tPPO, cPPO, and pPPO were 81.8%, 81.6%, and 74.9% after thermal processing at 50 °C for 60 min. It could be clearly seen that the semilog relationship between the residual activity of PPO from kudzu and treatment time followed first-order kinetics well, with the correlation coefficients (R^2^) ranging from 0.9708 to 0.9994. The *k* value of tPPO, cPPO, and pPPO at 50 °C increased successively and were 0.32 × 10^−2^, 0.36 × 10^−2^, and 0.52 × 10^−2^ min^−1^, respectively (Table 1). Similar results were observed in PPOs treated at 60, 70, and 80 °C (Figure 2b–d). With the increase in temperature, the difference during samples gradually became more obvious, and at 80 °C, the *k* value of tPPO, cPPO, and pPPO were 8.57 × 10^−2^, 14.63 × 10^−2^, and 23.67 × 10^−2^ min^−1^, respectively. It was observed that for tPPO, cPPO, and pPPO, the *k* value increased with the increase in processing temperature, indicating that kudzu PPO was more sensitive at higher temperatures. The highest *D* value (729.18 min) and *t*_1/2_ value (219.50 min) were found in tPPO at 50 °C, which also supports the viewpoints that higher temperature leads to lower stability, and tPPO was the most stable. In comparison with cPPO from other sources, the *D* values and *t*_1/2_ values were 73.99 and 22.29 min for bayberry [29], 39.0 and 11.8 min for mushroom [8], and 17.3 and 5.2 min for peach [30] at 60 °C, respectively, which are lower than 158.20 and 47.62 min in this study, indicating that PPO from kudzu was more thermostable than the above-mentioned PPO. The *Z* values of tPPO, cPPO, and pPPO were 21.25, 19.21, and 17.47 °C, and the corresponding *E_a_* were 102.68, 113.67, and 125.05 kJ/mol, respectively, the lower *Z* value and higher *E_a_* suggests an increased susceptibility to heat [8].

Thermodynamic parameters provide information on the enzyme stability in the thermal inactivation process, which was helpful for evaluating the possible effects of the stabilization or destabilization of the structure of an enzyme molecule and interactions between the enzyme and media [31]. Δ*H* is an indicator of the number of non-covalent bonds destroyed during the inactivation process [8]. The higher Δ*H* value of pPPO compared with that of tPPO and cPPO at the same temperature indicates that more non-covalent bonds were broken during the formation of transition states. When increasing the processing temperature, no significant changes of Δ*H* were noted for the three PPO samples, which suggests that the heat capacity of PPO did not change. Δ*G* is another important parameter related to the stability of the enzyme; a lower Δ*G* represents the lower stability of the enzyme [8] and a more spontaneous inactivation reaction [31]. Δ*S* is a physical quantity that reflects the degree of disorder of microscopic particles in the system. From 50 to 80 °C, the Δ*G* of tPPO, cPPO, and pPPO decreased, and the corresponding Δ*S* increased (with the exception of that of the tPPO at 65 and 70 °C and that of cPPO at 70 °C), which proves that the ordered structure and thermal stability of kudzu PPO decreased with the increasing temperature. Overall, the thermodynamics were closely related to the kinetics and unanimously showed that increasing the temperature led to a decrease in the stability of different PPO samples; at the same temperature, tPPO had the greatest resistance against heat followed by cPPO; pPPO was the most sensitive to heat.

These variations in inactivation kinetics and thermodynamic parameters of different PPO samples may be attributed to environmental conditions and food matrixes [26]. Ji et al. [31] observed that the *t*_1/2_ value of purified β-galactosidase (8.25 and 0.58 min at 55 and 60 °C, respectively) was significantly smaller than that of the crude enzyme (10.13 and 0.75 min) in the temperature range of 55–65 °C. Furthermore, Zhou et al. [11] found that pear PPO showed higher resistance to thermal inactivation in the food systems than in model systems. Food constituents in the food system or crude extract were rich and various, which might affect the thermal inactivation of the enzyme.

### 3.4. Effect of Food Constituents on the Activity of PPO

**Figure 3:**
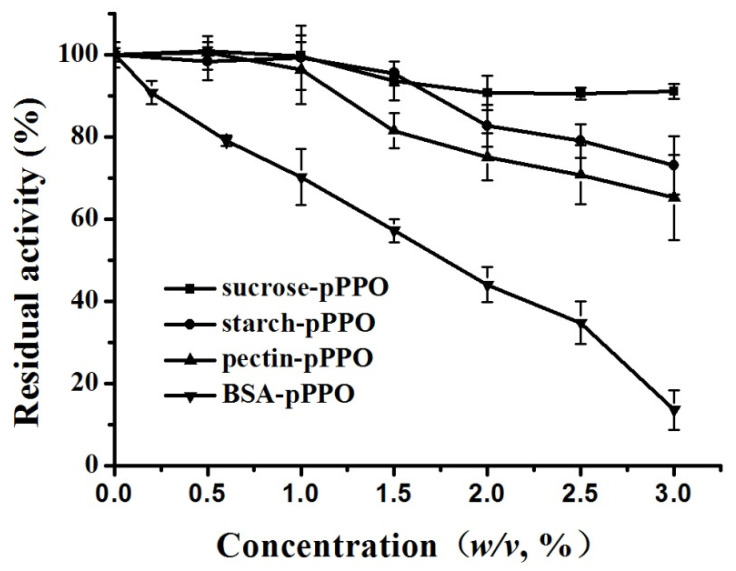
Effect of pectin, bovine serum albumin, starch and sucrose on the activity of purified PPO (pectin-pPPO, BSA-pPPO, starch-pPPO, and sucrose-pPPO) in concentrations ranging from 0.5% to 3% (*w*/*v*).

In order to further investigate the effect of food constituents on the activity of PPO, exogenous sucrose, starch, pectin, and BSA were added to pPPO (Figure 3). The results showed that different food constituents have different effects on PPO. BSA showed the greatest impact on PPO activity: the activity of PPO was significantly decreased to 70.2% as the concentration of BSA was 1.0% (*w*/*v*), and it was only 13.6% when the concentration of BSA increased to 3.0% (*w*/*v*). Compared with BSA, the effect of sucrose, starch, and pectin on pPPO activity was much smaller. For sucrose, starch, and pectin, PPO activity changed slightly when the concentration of constituents was lower than 1.0% (*w*/*v*). As the concentration increased, the effects of sucrose, starch, and pectin on PPO activity became distinct. The residual activities of sucrose-pPPO, starch-pPPO, and pectin-pPPO were 90.7%, 82.7%, and 75.1% when the additive amount reached 2.0%, and 86.6%, 63.2%, and 45.1% when the additive amount reached 3.0% (*w*/*v*). In general, all four food constituents displayed an inhibition effect on PPO activity, and BSA showed the strongest inhibition effect, followed by pectin and starch, and sucrose exhibited the weakest inhibition effect. Similarly, some previous reports studied the effects of food constituents on enzyme activity and found that different additives result in different influences on different enzymes. For instance, Isleroglu and Turker [32] reported saccharides (gum arabic and inulin) as coating materials to protect the microbial transglutaminase. Bayarri et al. [33] reported that the addition of 0–0.2% (*w*/*v*) pectin induced a decrease in lysozyme activity, and the minimum activity was found as the additive concentration of pectin reached 0.02%. Chisari et al. [34] found that 72.1% *D*-glucose and *D*-fructose treatment resulted in a decrease in the PPO activity of “Madame Moutot” strawberry fruit by ~50% and ~60%, respectively, while the addition of 1.8% *D*-glucose and *D*-fructose led to a slight increase in PPO activity. In this study, only the limited inhibition effect on PPO activity was observed with the addition of low molecular compound sucrose, while macromolecular compounds, such as BSA, pectin, and starch, showed a stronger inhibition effect on PPO.

According to the study by Matsue and Miyawaki [35], the catalytic ability of the enzyme may be largely related to water activity and solvent ordering, and Chakraborty et al. [21] reported that sugars could change water activity, which may be a reasonable explanation for the decrease in PPO activity caused by sucrose, starch, and pectin. In addition, the abundant hydroxyl groups in starch and pectin were conducive to the formation of hydrogen bonds, which may disrupt the equilibrium of the enzyme’s native structure in solvents and thus exhibit an inhibition effect [21]. Moreover, PPO can form a complex with pectin through a bridging mechanism, and the formation of electrostatic bridges may induce aggregation of PPO [36], which will hide the active site of the enzyme and lead to a lower substrate affinity and PPO activity. BSA exhibited the strongest inhibitory ability among the four food constituents. BSA molecule contains a free sulfhydryl group and 17 disulfide bonds that can be converted into sulfhydryl groups. The formed sulfhydryl groups can chelate and even remove the essential copper on the active sites of PPO, which was the factor that directly resulted in a decrease in PPO activity [37].

### 3.5. Effect of Food Constituents on the Thermal Inactivation Kinetics and Thermodynamics of PPO

**Figure 4:**
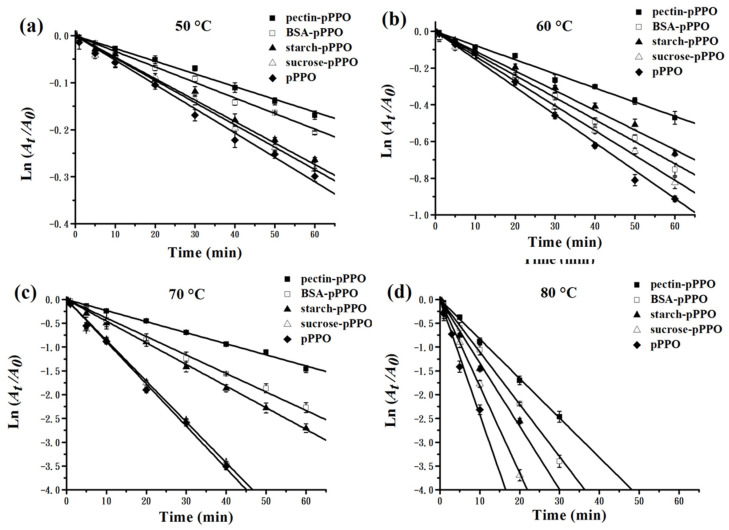
Thermal inactivation kinetics curves of purified PPO (pPPO) in the present of pectin (pectin-pPPO), bovine serum albumin (BSA-pPPO), starch(starch-pPPO), and sucrose (sucrose-pPPO) at a concentration of 1.0% (*w*/*v*) at 50 °C (**a**), 60 °C (**b**), 70 °C (**c**), and 80 °C (**d**) for 0–60 min.

Figure 4 presents the role of pectin, BSA, starch, and sucrose in the thermal inactivation of pPPO, and the thermal inactivation of pPPO, pectin-pPPO, BSA-pPPO, starch-pPPO, and sucrose-pPPO, fitting a linear relationship (*R*^2^ = 0.9721–0.9992). As shown in Figure 4a, pectin, BSA, starch, and sucrose reduced the thermal inactivation rate of pPPO, and the residual activities of pPPO, pectin-pPPO, BSA-pPPO, starch-pPPO, and sucrose-pPPO were 74.9%, 83.6%, 82.0%, 77.1%, and 75.1%, respectively, after thermal processing at 50 °C for 60 min, and the corresponding *k* values were 0.52 × 10^−2^, 0.27 × 10^−2^, 0.34 × 10^−2^, 0.44 × 10^−2^, and 0.48 × 10^−2^ min^−1^, respectively (Table 1). Similar results are shown in Figure 4b–d, and the corresponding *k* values of pectin-pPPO, BSA-pPPO, starch-pPPO, and sucrose-pPPO were 0.78 × 10^−2^, 0.89 × 10^−2^, 1.05 × 10^−2^, and 1.35 × 10^−2^ min^−1^ at 60 °C, 2.35 × 10^−2^, 3.63 × 10^−2^, 3.84 × 10^−2^ and 8.59 × 10^−2^ min^−1^ at 70 °C; and 8.32 × 10^−2^, 10.12 × 10^−2^, 14.34 × 10^−2^, and 21.97 × 10^−2^ min^−1^ at 80 °C, respectively. It can be noted that all four constituents exhibited protective effects on pPPO at all temperatures, and pectin showed the strongest protective effect, followed by BSA and starch, while sucrose showed the worst protection effect. These conclusions are also supported by gradually decreasing *D* values, *t*_1/2_ values, and *Z* values and the gradually increasing *E_a_* in the order of pectin-pPPO, BSA-pPPO, starch-pPPO, and sucrose-pPPO. As the thermal processing temperature increased, Δ*G* gradually decreased, Δ*S* gradually increased, and Δ*H* did not change significantly. These changes indicated that the stability of pectin-pPPO, BSA-pPPO, starch-pPPO, and sucrose-pPPO decreased with the increase in processing temperature. These results are also consistent with the results presented in Section 3.3, wherein cPPO and tPPO, which contain food constituents including pectin, protein, starch, and sucrose, had higher thermostability than that of pPPO. Similarly, Liu et al. [17] reported that pectin had a protective effect on the activity of peach PPO during thermal processing, and the protective effect was closely related to the concentration of pectin. Li et al. [19] found that pectin, BSA, sucrose, and trehalose could protect soluble acid invertase against HPP inactivation and the protective effect of sucrose and trehalose was weaker than that of pectin and BSA.

Generally, the stability of an enzyme is affected by hydrogen bonds, hydrophobic bonds, metal bonds, ionic interactions, and disulfide bridges [38]. PPO tends to form complexes with proteins or carbohydrates, and the combination of PPO hydrophobic clusters with protein or carbohydrate molecules can shield the hydrophobic area on its surface, thereby protecting the structure of PPO from unfolding and enhancing its resistance against thermal inactivation [17,19]. In addition, the hydrophobic interactions and hydrogen bonds established between PPO and these food constituents, as well as the increase in solution viscosity caused by the additives, may also improve the thermal stability of PPO.

### 3.6. Effect of Food Constituents on Tertiary Structure of PPO

**Figure 5:**
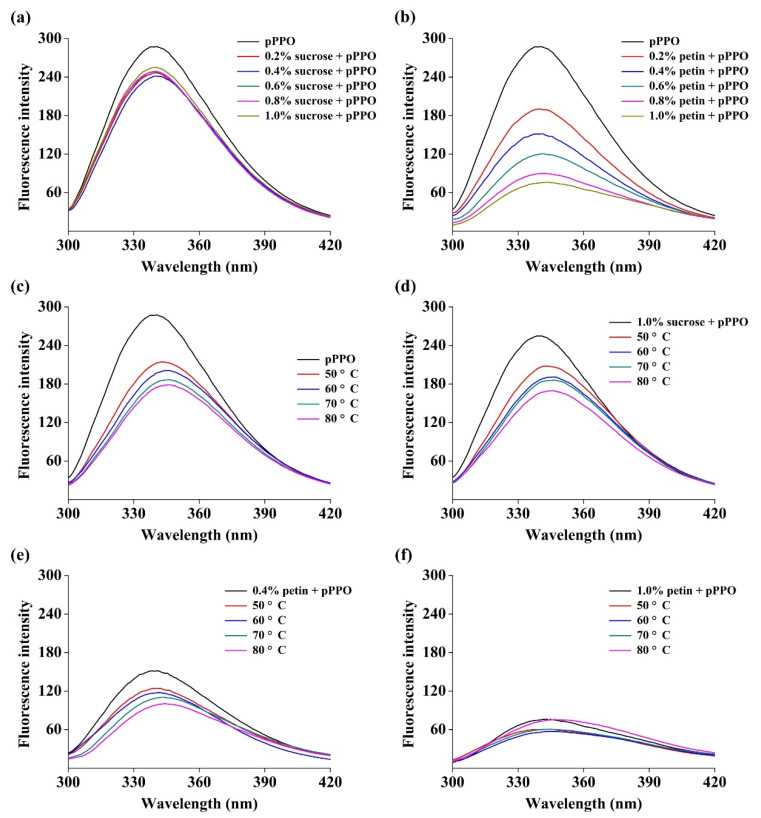
Fluorescence emission spectra of pPPO with sucrose (**a**) and pectin (**b**) at room temperature. From top to down, control sample (pPPO), pPPO with sucrose or pectin at the concentration of 0.2%, 0.4%, 0.6%, 0.8%, and 1.0% (*w*/*v*), respectively. Fluorescence emission spectra of pPPO (**c**), pPPO with sucrose (1.0%) (**d**), pPPO with pectin (0.4%) (**e**), and pPPO with pectin (1.0%) (**f**) during thermal treatment from room temperature (25 °C) to 80 °C.

The effects of pectin and sucrose on PPO tertiary structure were significantly different (Figure 5). Native pPPO displayed a 340.5 nm peak wavelength with a corresponding fluorescence intensity of 287.6, which is similar to values of mushroom PPO reported by Zhou et al. [27]. Sucrose with a concentration of 0.2–1.0% (*w*/*v*) did not significantly change the shape of pPPO fluorescence spectra (Figure 5a). Meanwhile 0.4%, 0.6%, 0.8%, and 1.0% (*w*/*v*) pectin caused a 0.5, 1.0, 1.5, and 2.0 nm redshift in the peak wavelength and a 47.3%, 58.1%, 68.7%, and 73.5% decrease, respectively, in the fluorescence intensity of pPPO (Figure 5b). The shape change in the fluorescence spectra was correlated with the conformational change in pPPO as well as the polarity change in the environment around tyrosine and tryptophan residues [39]. The more dramatic changes in pPPO in the presence of pectin rather than sucrose indicated that pectin had stronger interactions with pPPO, and the driving force may be the hydrogen bond, hydrophobic interaction, and electrostatic interaction. Moreover, the reduction in the environmental pH and hence proton transfer induced by pectin could be another reasonable explanation, according to Li et al. [19].

Thermal processing led to decreases in fluorescence intensity and a redshift in the peak wavelength of pPPO. The fluorescence intensities decreased to 74.5%, 69.9%, 64.8%, and 62.2%, with thermal processing at 50, 60, 70, and 80 °C, respectively, and the corresponding peak wavelengths were redshifted by 2.0, 4.0, 5.5, and 6.0 nm, respectively (Figure 5c). With the addition of 1.0% (*w*/*v*) sucrose, the redshifts of the pPPO fluorescence spectra were 2.5, 5.0, 6.0, and 5.5 nm at 50, 60, 70, and 80 °C (Figure 5d), which are very close to the values obtained in the absence of sucrose. In comparison, the peak wavelengths of pPPO were redshifted by 1.0, 1.0, 2.5, and 3.5 nm with 0.4% (*w*/*v*) pectin and 1.5, 2.0, 2.5, and 4.0 nm with 1.0% (*w*/*v*) pectin at 50, 60, 70, and 80 °C, respectively (Figure 5e,f), which are smaller than the values obtained in the case without pectin. These results illustrate that thermal processing could disrupt the tertiary structure of pPPO; however, the presence of pectin provided protection for the pPPO structure against thermal processing. This supports the results presented in Section 3.5, wherein pectin provided pPPO with stronger protection than of sucrose at all temperatures. Heating could unfold the pPPO molecules and expose them to amino acid residues [40], which may be conducive to the association of pPPO with pectin and hence improve its structural and thermal stability.

## 4. Conclusions

In this study, kudzu (*Pueraria lobata*) PPO with different constituent contents was successfully obtained by different purification procedures. It was noted that the thermal stability and inactivation rate constant of tPPO and cPPO containing food constituents was significantly higher than that of pPPO, confirming that food constituents were important factors leading to the differential thermal stability of PPO in the model and food systems. Food constituents pectin, BSA, starch, and sucrose exhibited a protective effect on pPPO against thermal processing, and the protective effect decreased orderly. Fluorescence spectrum results demonstrated that pectin could form stronger interactions with pPPO than sucrose and thus improve the structural and thermal stability of pPPO. In conclusion, the enzymatic reaction of PPO in the real food system is complex. This study provides a scientific and technological basis for the further evaluation of browning control and contributes to its practical application in foods.

## Figures and Tables

**Table 1:** Thermal inactivation parameters of PPO in kudzu tissue (tPPO), crude extract (cPPO), purification fractions (pPPO), and pPPO in the presence of food constituents ^1^.

Enzyme	Temperature (°C)	*k* (10^−2^ Min^−1^)	*D* min	*t*_1/2_ min	*E_a_* (kJ mol^−1^)	*Z* (°C)	∆*H* (kJ mol^−1^)	∆*G* (kJ mol^−1^)	∆*S* (J mol^−1^ K^−1^)
tPPO	50	0.32 ± 0.02	729.18 ± 46.66	219.50 ± 14.05	102.68 ± 2.18	21.25 ± 0.46	99.99 ± 2.18 ^c^	83.83 ± 0.17 ^abc^	50.02 ± 6.28 ^g^
	60	0.98 ± 0.02	235.02 ± 4.80	70.75 ± 1.44			99.91 ± 2.18 ^c^	83.37 ± 0.06 ^de^	49.63 ± 6.38 ^g^
	70	2.50 ± 0.04	92.12 ± 1.34	27.73 ± 0.40			99.82 ± 2.18 ^c^	83.29 ± 0.04 ^def^	48.19 ± 6.25 ^g^
	80	8.57 ± 0.34	26.90 ± 1.10	8.10 ± 0.33			99.74 ± 2.18 ^c^	82.18 ± 0.12 ^ij^	49.71 ± 6.32 ^g^
cPPO	50	0.36 ± 0.01	639.94 ± 17.78	192.64 ± 5.35	113.67 ± 0.86	19.21 ± 0.15	110.98 ± 0.86 ^b^	83.48 ± 0.07 ^cde^	77.88 ± 2.41 ^cde^
	60	1.46 ± 0.05	158.20 ± 5.43	47.62 ± 1.64			110.90 ± 0.86 ^b^	82.28 ± 0.10 ^ij^	81.05 ± 2.67 ^cd^
	70	3.50 ± 0.22	65.90 ± 4.15	19.84 ± 1.25			110.82 ± 0.86 ^b^	82.33 ± 0.18 ^ij^	80.66 ± 1.99 ^cd^
	80	14.63 ± 0.96	15.78 ± 1.05	4.75 ± 0.31			110.73 ± 0.86 ^b^	80.62 ± 0.19 ^l^	85.28 ± 2.91 ^c^
pPPO	50	0.52 ± 0.02	440.54 ± 18.96	132.62 ±5.71	125.05 ± 0.47	17.47 ± 0.09	122.36 ± 0.47 ^a^	82.48 ± 0.12 ^hi^	112.94 ± 1.52 ^b^
	60	1.54 ± 0.03	149.24 ± 3.08	44.93 ± 0.93			122.28 ± 0.47 ^a^	82.12 ± 0.06 ^ij^	113.73 ± 1.18 ^b^
	70	8.88 ± 0.65	26.03 ± 1.85	7.84 ± 0.56			122.20 ± 0.47 ^a^	79.68 ± 0.21 ^m^	120.3 9± 0.79 ^ab^
	80	23.67 ± 1.53	9.76 ± 0.63	2.94 ± 0.19			122.11 ± 0.47 ^a^	79.20 ± 0.19 ^n^	121.50 ± 1.83 ^ab^
Pectin- pPPO	50	0.27 ± 0.01	842.66 ± 17.58	253.67± 5.29	107.53 ± 1.78	20.29 ± 0.33	104.85 ± 1.78 ^bc^	84.22± 0.06 ^a^	58.41 ± 5.02 ^fg^
	60	0.78 ± 0.04l	297.02 ±15.93	89.41 ± 4.80			104.76 ± 1.78 ^bc^	84.02 ± 0.15 ^ab^	58.74 ± 5.15 ^fg^
	70	2.35 ± 0.06	98.16 ± 2.31	29.55 ± 0.70			104.68 ± 1.78 ^bc^	83.47 ± 0.07 ^cde^	60.06 ± 5.17 ^efg^
	80	8.32 ± 0.49	27.73 ±1.63	8.35 ± 0.49			104.60 ± 1.78 ^bc^	82.27 ± 0.17 ^ij^	63.22 ± 5.53 ^defg^
BSA- pPPO	50	0.34 ± 0.01	677.62 ± 19.94	203.98 ± 6.00	109.79 ± 2.60	19.90 ± 0.48	107.10 ± 2.60 ^b^	83.63 ± 0.08 ^bcd^	66.45 ± 7.16 ^defg^
	60	0.89 ± 0.04	258.11 ± 11.82	77.70 ± 3.56			107.02 ± 2.60 ^b^	83.63 ± 0.13 ^bcd^	66.23 ± 7.29 ^defg^
	70	3.63 ± 0.24	63.55 ±4.09	19.13 ±1.23			106.94 ± 2.60 ^b^	82.22 ± 0.19 ^ij^	69.97 ± 7.79 ^cdef^
	80	10.12 ± 0.46	22.79 ± 1.06	6.86 ± 0.32			106.85 ± 2.60 ^b^	81.70 ± 0.14 ^k^	71.23 ± 7.72 ^cdef^
Starch- pPPO	50	0.44 ± 0.01	519.62 ± 13.74	156.42 ± 4.14	110.94 ± 1.23	19.64 ± 0.22	108.25 ± 1.23 ^b^	82.92 ± 0.07 ^fg^	71.73 ± 3.29 ^cdef^
	60	1.05 ± 0.05	218.94 ± 10.49	65.91 ± 3.16			108.17 ± 1.23 ^b^	83.17 ± 0.13 ^ef^	70.77 ± 3.27 ^cdef^
	70	3.84 ± 0.06	59.92 ± 0.94	18.04 ± 0.28			108.09 ± 1.23 ^b^	82.06 ± 0.04 ^jk^	73.69 ± 3.58 ^cdef^
	80	14.34 ± 0.31	16.06 ± 0.35	4.84 ± 0.10			108.00 ± 1.23 ^b^	80.67 ± 0.06 ^l^	77.39 ± 3.53 ^cde^
Sucrose- pPPO	50	0.48 ± 0.01	483.11 ± 5.89	145.43 ± 1.77	129.34 ± 3.31	17.26 ± 0.25	126.65 ± 3.31 ^a^	82.72 ± 0.03 ^gh^	124.39 ± 9.45 ^ab^
	60	1.35 ± 0.02	171.00 ± 1.95	51.48 ± 0.59			126.57 ± 3.31 ^a^	82.49 ± 0.03 ^hi^	124.81 ± 9.43 ^ab^
	70	8.59 ± 0.24	26.83 ± 0.75	8.08 ± 0.23			126.49 ± 3.31 ^a^	79.77 ± 0.08 ^m^	132.29 ± 9.25 ^a^
	80	21.97 ±1.26	10.50 ± 0.59	3.16 ± 0.18			126.40 ± 3.31 ^a^	79.42 ± 0.17 ^mn^	133.04 ± 9.16 ^a^

^a^^–n^ Different lower-case letters within a row show statistically significant differences among values (*p* < 0.05). ^1^ Pectin, bovine serum albumin, starch and sucrose at the concentration of 1.0% (*w*/*v*) was added to pPPO solution (pectin-pPPO, BSA-pPPO, starch-pPPO, and sucrose-pPPO). *k* is the inactivation rate constant (min^−1^). *D* is the decimal reduction time. *t*_1/2_ is the half-life value. *E*_*a*_ is the activation energy (kJ/mol). *Z* is the temperature needed to reduce the *D* value by one log cycle. Δ*H* is change in enthalpy. Δ*G* is Gibbs free energy. Δ*S* is entropy.

## Data Availability

The data presented in this study are available in this article.

## Supplementary Materials

The following are available online at https://www.mdpi.com/article/10.3390/foods10061320/s1. Table S1: Purification of PPO from kudzu; Table S2: Change of constituent content in kudzu tissue, crude PPO (cPPO) solution, and purified PPO (pPPO) solution; Figure S1: SDS-PAGE electrophoresis of the purified PPO; Figure S2: Effect of pH on PPO activity (a) and Lineweaver–Burk plots of PPO (b).
