# Supplementary material for: Thermal Inactivation Kinetics of Kudzu (Pueraria lobata) Polyphenol Oxidase and the Influence of Food Constituents
Source: Foods. 2021 Jun 8;10(6):1320. doi: 10.3390/foods10061320 (PMC8226850; doi:10.3390/foods10061320)
Supplement: Supplementary file 1 [file foods-10-01320-s001.zip › foods-1240769-supplementary.pdf]

Supplementary Material

Thermal Inactivation Kinetics of Kudzu (*Pueraria lobata*) Polyphenol Oxidase and the Influence of Food Constituents

Table S1. Purification of PPO from kudzu

Purification step	Total activity (U)	Total protein (mg)	Specific activity (U/mg)	Recovery (%)	Purification (fold)
Crude extract	131,333 ± 1154	148.2 ± 2.4	886 ± 22	100.0 ± 0.9	1.0 ± 0.02
$(NH_4)_2SO_4$ precipitation	63,135 ± 852	13.9 ± 0.1	4,553 ± 71	48.1 ± 0.6	5.1 ± 0.1
DEAE sepharose fast flow column	43,200 ± 216	1.3 ± 0.04	34,001 ± 1123	32.9 ± 0.2	38.4 ± 1.3

Table S2. Change of constituent content in kudzu tissue, crude PPO (cPPO) solution, and purified PPO (pPPO) solution

	Starch	Protein	Pectin	Sucrose
Kudzu tissue (/100g tissue)	21.42 ± 1.23	1.42 ± 0.18	1.19 ± 0.13	1.57 ± 0.07
cPPO solution (/100g of solution)	0.37 ± 0.05	0.71 ± 0.04	0.51 ± 0.02	0.58 ± 0.09
pPPO solution (/100g of solution)	–[a]	0.11 ± 0.02	–	–

[a] –, not detected.

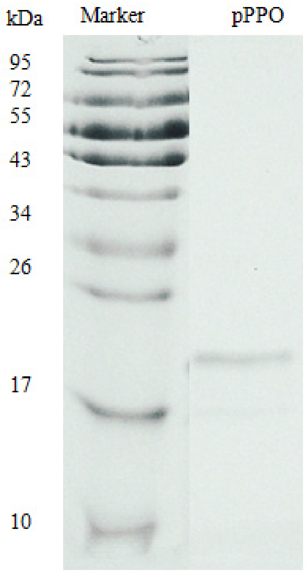

Figure S1. SDS-PAGE electrophoresis of the purified PPO

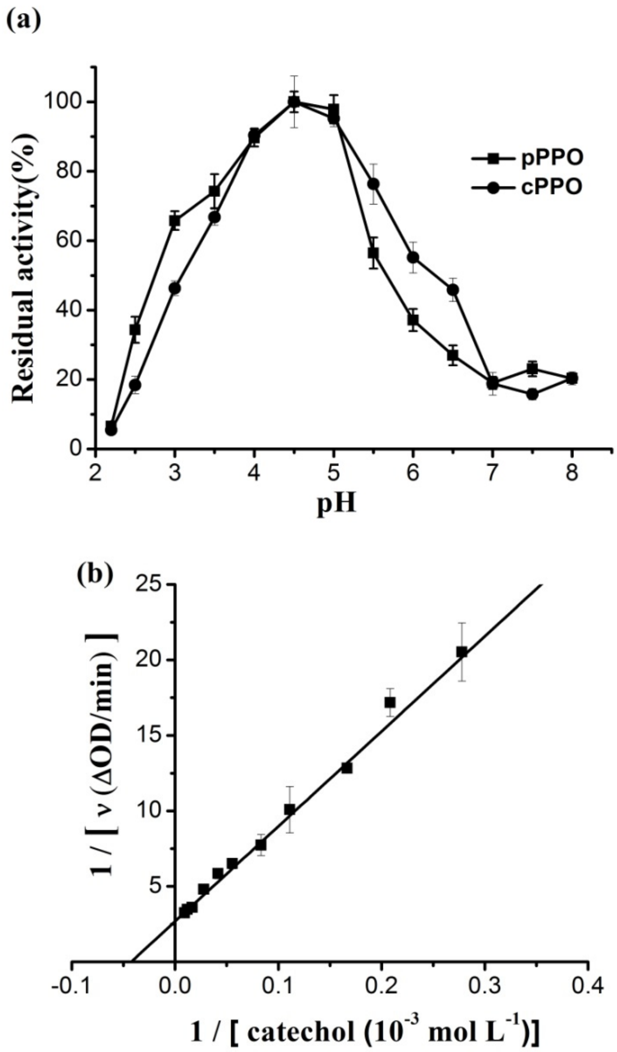

Figure S2. Effect of pH on PPO activity (a) and Lineweaver-Burk plots of PPO (b)
